# High-density genotyping reveals signatures of selection related to acclimation and economically important traits in 15 local sheep breeds from Russia

**DOI:** 10.1186/s12864-019-5537-0

**Published:** 2019-05-08

**Authors:** Andrey A. Yurchenko, Tatiana E. Deniskova, Nikolay S. Yudin, Arsen V. Dotsev, Timur N. Khamiruev, Marina I. Selionova, Sergey V. Egorov, Henry Reyer, Klaus Wimmers, Gottfried Brem, Natalia A. Zinovieva, Denis M. Larkin

**Affiliations:** 1 The Federal Research Center Institute of Cytology and Genetics, The Siberian Branch of the Russian Academy of Sciences (ICG SB RAS), Novosibirsk, Russia; 2 L.K. Ernst Federal Science Center for Animal Husbandry, Podolsk, 142132 Russia; 3 Novosibirsk State University, Novosibirsk, 630090 Russia; 4 Research Institute of Veterinary Medicine of Eastern Siberia, The Branch of the Siberian Federal Scientific Center for Agrobiotechnologies of the Russian Academy of Sciences, Chita, Russia; 5 All-Russian Research Institute of Sheep and Goat Breeding - branch of the Federal State Budgetary Scientific Institution North Caucasian Agrarian Center, Stavropol, 355017 Russia; 6 Siberian Research Institute of Animal Husbandry, Krasnoobsk, Russia; 7 Institute of Genome Biology, Leibniz Institute for Farm Animal Biology (FBN), Dummerstorf, Germany; 8 Institute of Animal Breeding and Genetics, University of Veterinary Medicine, Vienna, Austria; 9 Royal Veterinary College, University of London, London, UK

**Keywords:** Local sheep breeds, Selection, Adaptation, Genotyping, Russian Federation

## Abstract

**Background:**

Domestication and centuries of selective breeding have changed genomes of sheep breeds to respond to environmental challenges and human needs. The genomes of local breeds, therefore, are valuable sources of genomic variants to be used to understand mechanisms of response to adaptation and artificial selection. As a step toward this we performed a high-density genotyping and comprehensive scans for signatures of selection in the genomes from 15 local sheep breeds reared across Russia.

**Results:**

Results demonstrated that the genomes of Russian sheep breeds contain multiple regions under putative selection. More than 50% of these regions matched with intervals identified in previous scans for selective sweeps in sheep genomes. These regions contain well-known candidate genes related to morphology, adaptation, and domestication (e.g., *KITLG, KIT, MITF,* and *MC1R*), wool quality and quantity (e.g., *DSG@*, *DSC@*, and *KRT@*), growth and feed intake (e.g., *HOXA@, HOXC@, LCORL, NCAPG, LAP3,* and *CCSER1*), reproduction (e.g., *CMTM6, HTRA1, GNAQ, UBQLN1,* and *IFT88*), and milk-related traits (e.g., *ABCG2, SPP1, ACSS1,* and *ACSS2*). In addition, multiple genes that are putatively related to environmental adaptations were top-ranked in selected intervals (e.g., *EGFR, HSPH1, NMUR1*, *EDNRB*, *PRL, TSHR,* and *ADAMTS5*). Moreover, we observed that multiple key genes involved in human hereditary sensory and autonomic neuropathies, and genetic disorders accompanied with an inability to feel pain and environmental temperatures, were top-ranked in multiple or individual sheep breeds from Russia pointing to a possible mechanism of adaptation to harsh climatic conditions.

**Conclusions:**

Our work represents the first comprehensive scan for signatures of selection in genomes of local sheep breeds from the Russian Federation of both European and Asian origins. We confirmed that the genomes of Russian sheep contain previously identified signatures of selection, demonstrating the robustness of our integrative approach. Multiple novel signatures of selection were found near genes which could be related to adaptation to the harsh environments of Russia. Our study forms a basis for future work on using Russian sheep genomes to spot specific genetic variants or haplotypes to be used in efforts on developing next-generation highly productive breeds, better suited to diverse Eurasian environments.

**Electronic supplementary material:**

The online version of this article (10.1186/s12864-019-5537-0) contains supplementary material, which is available to authorized users.

## Background

The domestication and selective breeding of livestock species has led to changes in their genomes to meet the needs of humans and adapting livestock to various environments [1]. Many centuries of artificial selection shaped the genomes of contemporary sheep breeds by selective sweeps, which are associated with a variety of economically important traits, such as quality and quantity of wool, milk and meat. On the other hand, the history and development of these breeds is associated with the history of human migrations [2, 3]. After domestication about 9000–11,000 years ago on the territory of modern Iran [4], sheep have been spread worldwide, accompanying human migrations, e.g., the nomad’s expansion [2–4]. Thus, both artificial selection and acclimatization to various environments have contributed to the diversity of local breeds, expressing distinct and sometimes contrasting phenotypes (e.g., polled and horned breeds, black and white fleece, breeds adapted to the hot temperatures in Africa and the cold climates of Siberia). Therefore, domestic sheep should be considered as a valuable model species to study genome reaction to both environmental adaptation and artificial selection.

Searches for genome intervals, genes and polymorphisms which determine performance for economically important traits in sheep have resulted in revealing signatures of selection likely associated with coat colour [5–7], tail fat deposition [8], muscle growth [9], milk yield [10], reproductive traits [6, 11], wool [12], resistance to parasites [13], presence/absence of horns [6, 11], etc. These chromosome regions contain markers (genes or regulatory sequences) which should be the focus of future efforts on the improvement of local and multinational breeds using the rapidly-developing plethora of contemporary genetics tools.

Sheep have been successfully bred in different environments including some harsh ones, suggesting that the process of extreme acclimation could also lead to selective sweeps in the genomes of local breeds. Environment-influenced adaptations are complex, effecting multiple biochemical processes; therefore signatures of selection would be expected in genes from different pathways [14]. Thus, adaptation to high altitudes is an essential feature for the sheep breeding industry in countries with a predominating mountain terrain. Genomic studies of Tibetan sheep identified several candidate genes, associated with tolerance to hypoxia (e.g., *EPAS1, CRYAA, LONP1, NF1, DPP4, SOD1, PPARG,* and *SOCS2*). It was proposed that a key gene for adaptation to hypoxia is the *EPAS1*, which effects the mean corpuscular haemoglobin concentration and mean corpuscular volume [15]. A study of sheep breeds from a different region, the Himalayas, suggests that the *FGF-7* is a candidate for protection against pulmonary injuries and affects efficiency of lungs in sheep that inhabit high-altitude areas [16].

Temperature regime is one of the key climate factors that influence survival and successful breeding of livestock species. Multiple genes and gene networks are affected in different environments. For instance, Kim et al. (2016) shows that in the genomes of Egyptian indigenous sheep, there are changes in multiple genes that influence adaptation to hot arid environments including genes involved in melanogenesis, body size and development, energy and digestive metabolism, as well as in nervous and autoimmune response [17]. Furthermore, Lv and co-workers (2014) identified 17 genes putatively associated with climate-driven selection when they looked at a set of native sheep populations from a worldwide range of geographic areas [18]. Nine of the genes were directly involved in energy and regulation activities (e.g., *TBC1D12* and *FBXO8*) or encoded enzyme activators (e.g., *THY1*), while eight additional genes were involved in endocrine and autoimmune processes (e.g., *EDNRB*, *NMUR1*, *PRL*, *IL12RB1*, and *ACVR2A*)*.* Signatures of adaptations, caused by temperature and sunlight, were linked to the *TBC1D12* [18]. Comparative genomics provides additional evidence for links between genes and acclimation. For instance, *TRPM8* (transient receptor potential cation channel subfamily M member 8) plays a role in thermal sensation in mice [19]. The same gene was linked to cold tolerance in sheep [6, 7, 11]. These examples demonstrate that different genes could be affected by selection to similar environments, suggesting that studies of multiple breeds adapted to a variety of conditions could help revealing a full set of genomic regions affected during the process of adaptation.

As a step toward this goal, we present the scan for signatures of selection in a set of 15 Russian local sheep breeds. For our current analysis we chose a subset of genetically different breeds and breeds adapted to cold climates of Siberia based on results of our previous study focused on the phylogenetic history of 25 Russian sheep breeds, performed using a medium-density ovine SNP array [20]. We added one additional breed from Siberia (putatively adapted to cold climate), to extend the dataset of cold-adapted breeds. Due to its large area and geographical position, Russia comprises a variety of diverse climatic zones present in Eurasia. Nevertheless, about three quarters of Russia is represented by territories in a continental climate with occasionally extremely low winter temperatures (- 30 °C and below). Because of adaptation to the diverse environments of Russia, we expect that the analysis of Russian native breeds will: a) confirm the previously reported signatures of selection, and b) point to new regions not found in other studies, which could be important for the local adaptations especially in the northern regions. We used a combination of two powerful approaches to identify signatures of selection, one based on differences in frequencies of haplotypes in sets of related breeds (hapFLK), and the second one combining various breed-specific selection statistics into a single integrated framework (DCMS). Together these methods provide a complimentary way of finding signatures of selection in the set of 15 Russian sheep breeds, which we genotyped on a high-density SNP array. We analysed a minimum of 40 autosomes per breed (20 individuals) to ensure detection of the most common signatures of selection by both methods. Our data represent the first comprehensive analysis of selective sweeps in the genomes of Russian local sheep breeds. Our results could be used by the international community to better understand genetic mechanisms of adaptation to various environments, and by breeders looking to develop new breeds that are better adapted to local conditions or to improve adaptation in extant breeds.

## Results

### Breed groups

**Fig. 1:**
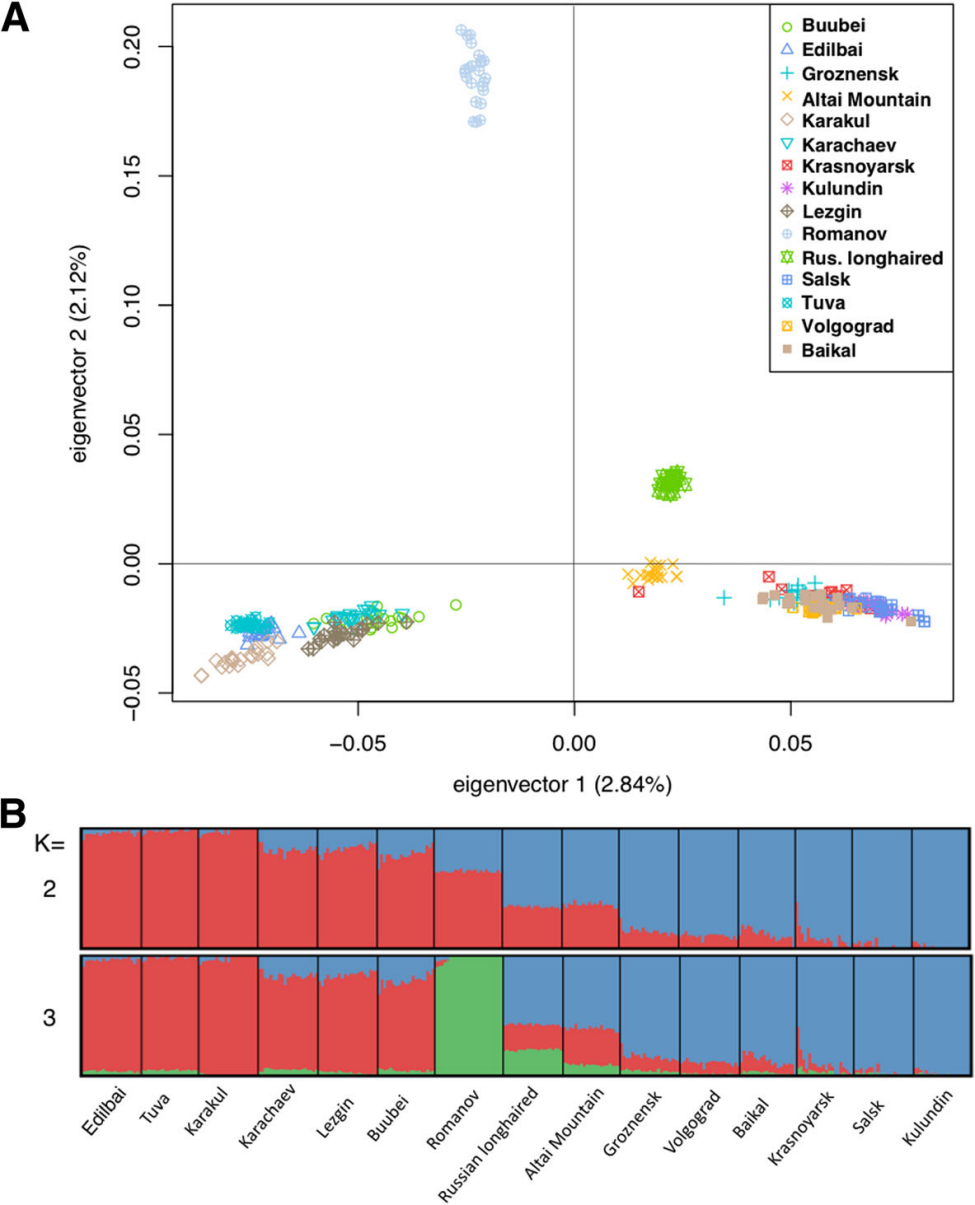
PCA (**a**) and ADMIXTURE (**b**) analysis indicate clear separation of the two major sheep groups. The GROUP1 includes Edilbai, Tuva, Karakul, Karachaev, Lezgin, Buubei, Romanov breeds and the GROUP2 includes Russian Longhaired, Altai Mountain, Groznensk, Volgograd, Baikal, Krasnoyarsk, Salsk, and Kulundin breeds

The Admixture and Principal Component Analysis (PCA; PC1) suggested the presence of two well-differentiated clusters of breeds in our dataset (Fig. 1): Buubei, Lezgin, Karachaev, Karakul, Tuva, Edilbai, Romanov (GROUP1) and the Russian Longhaired, Altai Mountain, Groznensk, Salsk, Volgograd, Krasnoyarsk, Baikal, and Kulundin (GROUP2). Although the Romanov breed demonstrated a substantial differentiation from the rest of the breeds we assigned it to GROUP1 because it had a higher GROUP1 component in the ADMIXTURE (K = 2) analysis, clustered with other GROUP1 breeds based on the PCA PC1 results, and additionally represents a coarse wool breed (as all other breeds from the GROUP1). In total, 312 animals from 15 Russian local sheep breeds with a mean number of 21 individuals per breed were used in these analyses (Table 1).

**Table 1:** Breeds and breed groups

Breed	No. samples	Wool type	Reference	Breed group
Buubei	20	coarse	[20]	GROUP1
Lezgin	21	coarse	[20]	GROUP1
Karachaev	21	coarse	[20]	GROUP1
Karakul	21	coarse	[20]	GROUP1
Tuva	20	coarse	[20]	GROUP1
Edilbai	21	coarse	[20]	GROUP1
Romanov	24	coarse	[20]	GROUP1
Russian Longhaired	21	semi-fine	[20]	GROUP2
Altai Mountain	20	semi-fine	[20]	GROUP2
Groznensk	21	fine	[20]	GROUP2
Salsk	21	fine	[20]	GROUP2
Volgograd	21	fine	[20]	GROUP2
Krasnoyarsk	20	fine	[107]	GROUP2
Baikal	20	fine	[20]	GROUP2
Kulundin	20	fine	[20]	GROUP2
Average/Total	21/312	–	–	–

**Fig. 2:**
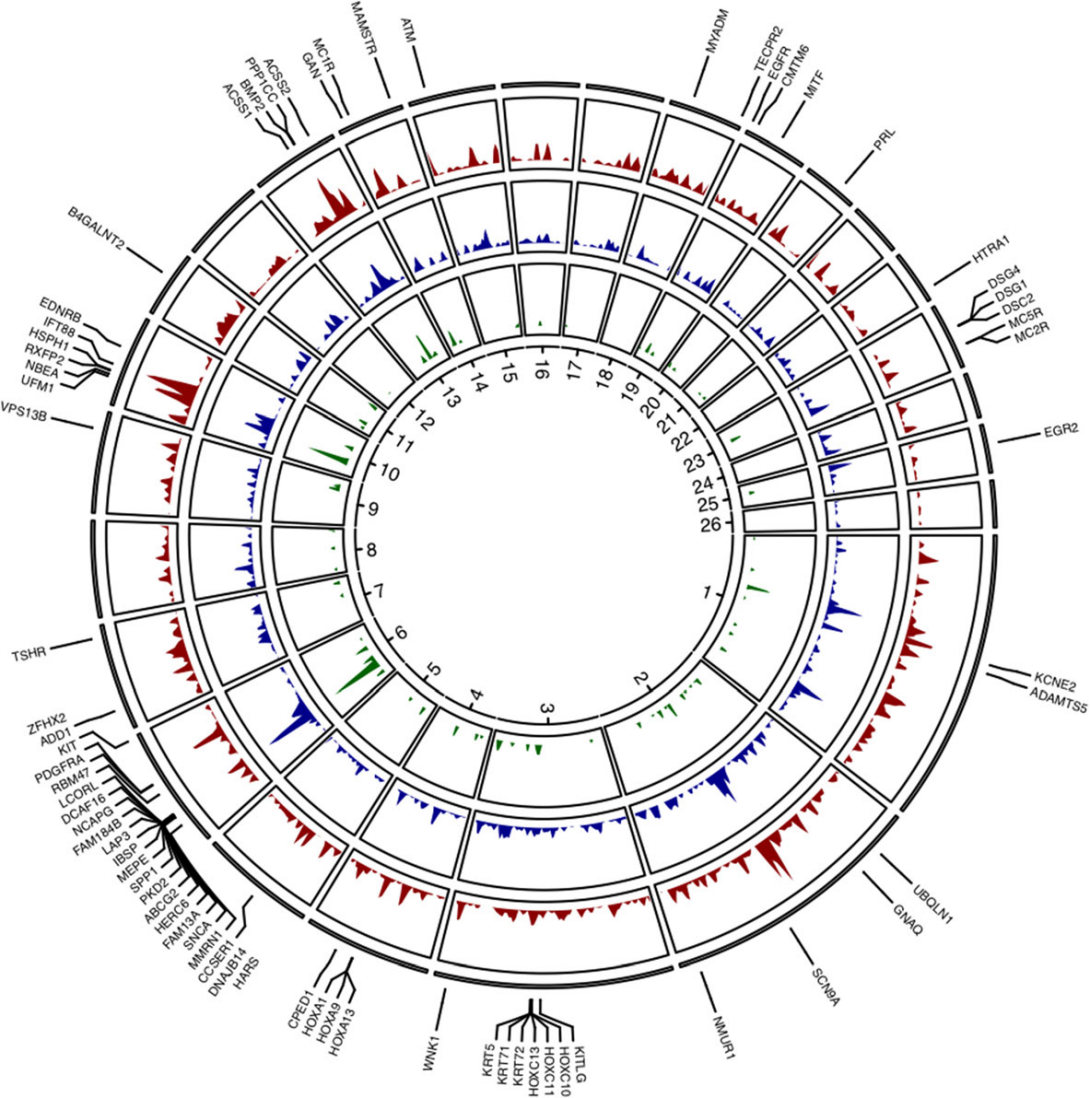
Circus plot of the relative density of selected regions along the ovine genome. DCMS statistics corresponding to GROUP1 is in dark red, to GROUP2 in dark blue, and hapFLK statistics for all breeds shown in green

The de-correlated composite of multiple signals (DCMS) and hapFLK statistics for individual breeds and groups of breeds overlapped to some extent: 546 DCMS-detected regions, covering 100.8 Mbp of the sheep genome sequence were found in shared intervals, providing independent support for selected regions (Fig. 2, see Additional file 1). However, because the hapFLK statistic detects signatures of selection within groups of breeds and the DCMS in our study was used to combine statistics within a breed, the hapFLK results could not be added to the DCMS framework. The hapFLK revealed additional selective sweeps within groups of breeds missed by the DCMS, while the DCMS was efficient in detecting narrower selective sweeps often attributed to individual breeds.

### Composite measure of selection

DCMS statistics were calculated for each single nucleotide polymorphism (SNP) for each breed. After fitting for normal distribution, calculation of *p*-values and correction for multiple testing, we obtained from 134 to 238 genomic intervals under putative selection per breed (q-value < 0.01) with a total of 3069 regions detected across all breeds (with some overlaps between breeds; Fig. 2, see Additional file 2). The size of the genomic regions putatively under selection varied from 1 bp to 3,510,819 bp, with the average size equal to 120,924 bp. The total number of genes across all selected regions per breed ranged from 146 to 366.

### HapFLK

**Fig. 3:**
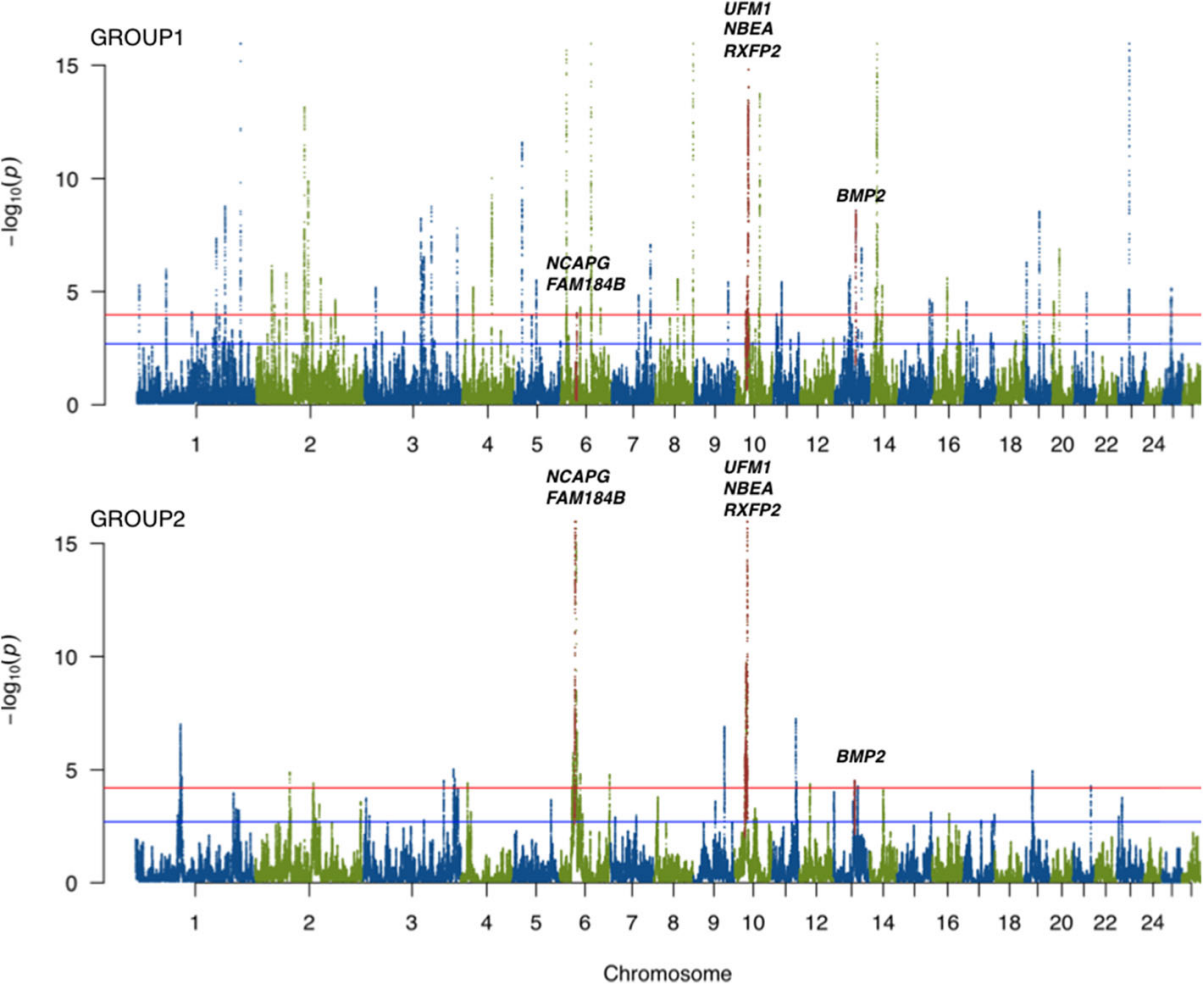
Manhattan plots of the hapFLK statistics for two groups of Russian sheep breeds. The shared signatures of selection between the two groups are highlighted in red and the candidate genes names are indicated. Blue and red horizontal lines indicate suggestive (q-value< 0.05) and significant (q-value< 0.01) FDR thresholds, respectively

The total number of selected regions identified by the hapFLK analysis (122; Fig. 3, see Additional file 2) was lower than found by the DCMS method. The largest number of hapFLK-detected regions was observed in the GROUP1 set of breeds (62), followed by the all-breed (33) and the GROUP2 (27) sets. The GROUP1 and GROUP2 shared five common hapFLK intervals. One region was found on ovine chromosome (OAR) 6 (34.6–37.5 Mbp) containing multiple genes with known effects on economically important traits such as milk production and growth. Three overlapping regions were detected on this chromosome: one near the *UFM1* gene involved in brain development and abnormalities in humans [21]; the second, near neurobeachin (*NBEA*), a gene previously associated with autism in humans [22] and wool production traits in sheep [23]; and the last, near *RXFP2*, involved in formation of horns in sheep [24] and cattle [25]. The last overlapping region was found on OAR13 and contained *BMP2,* the only common gene between the groups in this region. *BMP2* is associated with the body size and developmental traits in sheep [17]. Sizes of the hapFLK putatively selected regions ranged from 70 Kbp to 5.7 Mbp with an average size of 667.7 Kbp.

### Candidate genes for adaptation of the Russian sheep breeds to environmental and climate challenges

In the regions under putative selection we looked for genes that could be related to adaption of Russian sheep breeds to local environments (Table 2). We noticed that five breeds: Karachaev, Krasnoyarsk, Lezgin, Salsk, and Altai Mountain had a common signature of selection reported by the DCMS method near the *ZFHX2* (zinc finger homeobox 2), a gene which was shown to be responsible for an inability to feel pain in humans (including low and high temperatures) [26]. The same authors report that *ZFHX2* knock-out mice had significantly higher acute thermal pain thresholds [26]. Interestingly, several other genes directly involved in hereditary sensory and autonomic neuropathies [27] often accompanied with inability to feel pain and cold temperatures in humans were found top-ranked in selected regions in the Russian sheep breeds. Among them were the *SCN9A* [28], reported for Buubei, *WNK1* [29] reported for Salsk, *HARS* [30] reported for Karakul and Kulundin breeds, *TECPR2* [31] reported for Tuva sheep, *GAN* [27] for Karachaev, and *EGR2* [27] for GROUP1.

**Table 2:** Selective sweeps and candidate genes related to acclimation and economically important traits found in genomes of Russian sheep breeds

OAR	Start (Mbp)	End (Mbp)	q-value	Breed/Group	Method	No. genes	Candidate genes (rank)	Trait
1	119.12	119.49	1.90E-09	Karachaev	DCMS	6	KCNE2 (1)	growth
1	119.09	119.49	7.67E-05	Karakul	DCMS	6	KCNE2 (1)	growth
1	119.23	119.45	0.001775722	Tuva	DCMS	5	KCNE2 (1)	growth
1	127.64	127.75	0.000366751	Altai Mountain	DCMS	1	ADAMTS5 (1)	acclimation
1	127.63	127.74	0.000199884	Baikal	DCMS	1	ADAMTS5 (1)	acclimation
1	127.63	127.69	0.007036415	Kulundin	DCMS	1	ADAMTS5 (1)	acclimation
2	35.41	36.06	0.000153169	GROUP1	hapFLK	6	UBQLN1 (5)	reproduction
2	35.87	35.92	0.004399798	Salsk	DCMS	1	UBQLN1 (1)	reproduction
2	58.59	58.75	0.000834252	Edillbai	DCMS	1	GNAQ (1)	reproduction
2	58.62	58.74	0.002702535	Lezgin	DCMS	1	GNAQ (1)	reproduction
2	58.64	58.71	0.008858869	Karakul	DCMS	1	GNAQ (1)	reproduction
2	142.19	142.30	2.19E-05	Buubei	DCMS	1	SCN9A (1)	temperature sensation
2	232.36	232.41	0.00631797	Romanov	DCMS	1	NMUR1 (1)	acclimation
3	124.57	124.69	0.000275686	Edillbai	DCMS	1	KITLG (1)	coat colour/domestication
3	124.55	124.68	8.86E-05	Buubei	DCMS	1	KITLG (1)	coat colour/domestication
3	132.24	132.94	9.65E-05	GROUP1	hapFLK	16	HOXC@ (1–7, 9)	growth
3	132.26	132.53	6.51E-07	Lezgin	DCMS	9	HOXC@ (1–6, 8,9)	growth
3	132.31	132.51	0.000416517	Karakul	DCMS	9	HOXC@ (1–6,8,9)	growth
3	132.31	132.42	0.008497984	Edillbai	DCMS	7	HOXC@ (1–6)	growth
3	133.58	133.77	9.72E-13	Salsk	DCMS	8	KRT@ (1–8)	wool
3	133.59	133.76	1.36E-07	Krasnoyarsk	DCMS	7	KRT@ (1–7)	wool
3	133.60	133.75	3.73E-07	Groznensk	DCMS	6	KRT@ (1–6)	wool
3	133.56	133.75	3.24E-10	Baikal	DCMS	6	KRT@ (1–6)	wool
3	133.57	133.72	1.35E-08	Kulundin	DCMS	5	KRT@ (1–5)	wool
3	133.61	133.66	0.005739761	Volgograd	DCMS	1	KRT72 (1)	wool
3	211.49	211.62	0.000569719	Salsk	DCMS	1	WNK1 (1)	temperature sensation
4	68.29	69.18	4.31E-08	GROUP1	hapFLK	12	HOXA@ (1–9)	growth
4	68.81	69.17	6.70E-06	Karakul	DCMS	8	HOXA@ (1–8)	growth
4	68.75	69.13	3.56E-08	Buubei	DCMS	10	HOXA@ (1–9)	growth
4	68.64	68.99	1.25E-06	Russian Longhaired	DCMS	10	HOXA@ (2–9)	growth
4	68.62	68.97	9.34E-14	Karachaev	DCMS	10	HOXA@ (1,3–10)	growth
4	68.62	68.92	5.18E-05	Kulundin	DCMS	4	HOXA@ (2–4)	growth
4	85.61	85.75	3.38E-07	Karachaev	DCMS	1	CPED1 (1)	growth
4	85.66	85.71	0.000339136	Krasnoyarsk	DCMS	1	CPED1 (1)	growth
4	85.67	85.71	0.002455069	Edillbai	DCMS	1	CPED1 (1)	growth
4	85.67	85.70	0.002368717	Altai Mountain	DCMS	1	CPED1 (1)	growth
5	49.07	49.38	0.000134196	Karakul	DCMS	12	HARS (1)	temperature sensation
5	49.19	49.27	0.001308813	Kulundin	DCMS	4	HARS (1)	temperature sensation
6	24.71	25.01	1.33E-08	Kulundin	DCMS	3	DNAJB14 (3)	reproduction
6	24.71	25.01	4.37E-09	Baikal	DCMS	3	DNAJB14 (1)	reproduction
6	24.69	24.98	0.000102818	Salsk	DCMS	3	DNAJB14 (1)	reproduction
6	24.71	24.97	6.53E-09	Krasnoyarsk	DCMS	3	DNAJB14 (1)	reproduction
6	24.74	24.95	0.000157066	Altai Mountain	DCMS	3	DNAJB14 (1)	reproduction
6	24.74	24.94	8.54E-05	Lezgin	DCMS	3	DNAJB14 (1)	reproduction
6	24.75	24.90	0.005804894	Edillbai	DCMS	1	DNAJB14 (1)	reproduction
6	24.71	24.89	0.000171107	Tuva	DCMS	2	DNAJB14 (1)	reproduction
6	24.65	24.88	6.15E-06	Buubei	DCMS	1	DNAJB14 (1)	reproduction
6	24.71	24.88	0.000392132	Karakul	DCMS	1	DNAJB14 (1)	reproduction
6	24.71	24.86	0.000596432	Karachaev	DCMS	1	DNAJB14 (1)	reproduction
6	34.63	40.33	0	GROUP2	hapFLK	25	MEPE (1) IBSP (2) SPP1 (3) PKD2 (4) ABCG2 (5) LAP3 (6) MED28 FAM184B (8) NCAPG (11) HERC6 (12) LCORL (13) FAM13A (19) SNCA (22)	growth, milk
6	35.67	39.19	8.04E-10	Volgograd	DCMS	19	HERC6 (1) ABCG2 (8) PKD2 (10) SPP1 (11) MEPE (12) IBSP (13) LAP3 (14) FAM184B (16) NCAPG (18) LCORL (19)	growth, milk
6	34.85	38.82	0	All	hapFLK	21	LCORL (1) NCAPG (2) FAM184B (4) LAP3 (6) IBSP (7) MEPE (8) SPP1 (9) PKD2 (10) ABCG2 (11) HERC6 (13) FAM13A (19)	growth, milk
6	36.40	38.62	3.50E-08	Kulundin	DCMS	11	LAP3 (2) FAM184B (3) NCAPG (5) LCORL (6) IBSP (7) MEPE (8) SPP1 (9) PKD2 (10) ABCG2 (11)	growth, milk
6	36.87	38.28	3.13E-11	Baikal	DCMS	6	LAP3 (1) FAM184B (2) NCAPG (4) LCORL (5)	growth
6	36.52	38.24	3.68E-08	Salsk	DCMS	10	LAP3 (2) FAM184B (3) NCAPG (5) LCORL (6) IBSP (7) MEPE (8) SPP1 (9) PKD2 (10) ABCG2 (11)	growth, milk
6	36.45	37.87	5.07E-07	Krasnoyarsk	DCMS	11	PKD2 (1) SPP1 (2) ABCG2 (3) MEPE (4) IBSP (5) LAP3 (6) FAM184B (7) NCAPG (9) LCORL (10)	growth, milk
6	37.23	37.63	3.10E-06	Lezgin	DCMS	4	LCORL (1) NCAPG (2) FAM184B (4)	growth
6	37.36	37.60	0.000701655	Edillbai	DCMS	1	LCORL (1)	growth
6	37.25	37.45	0.008575203	GROUP1	hapFLK	3	NCAPG (1) FAM184B (3)	growth
6	37.35	37.44	0.007856497	Buubei	DCMS	1	LCORL (1)	growth
6	37.09	37.14	0.009452078	Altai Mountain	DCMS	2	LAP3 (1)	growth
6	36.52	36.80	7.87E-05	Baikal	DCMS	3	SPP1 (1) PKD2 (2) ABCG2 (3)	milk
6	36.09	36.51	5.45E-05	Karakul	DCMS	5	HERC6 (1)	growth
6	36.09	36.45	4.06E-07	Lezgin	DCMS	6	HERC6 (2)	growth
6	36.11	36.40	0.000168675	Kulundin	DCMS	4	HERC6 (4)	growth
6	36.05	36.33	0.000432111	Krasnoyarsk	DCMS	5	HERC6 (4)	growth
6	36.23	36.30	0.003706257	Altai Mountain	DCMS	1	HERC6 (1)	growth
6	36.16	36.30	0.000321255	Salsk	DCMS	4	HERC6 (4)	growth
6	34.87	35.07	1.56E-05	Kulundin	DCMS	1	SNCA (1)	milk
6	34.87	35.03	0.000133556	Volgograd	DCMS	1	SNCA (1)	milk
6	34.89	35.02	0.000295081	Krasnoyarsk	DCMS	1	SNCA (1)	milk
6	34.90	35.02	0.000209331	Baikal	DCMS	1	SNCA (1)	milk
6	34.29	34.84	0.003646083	All	hapFLK	1	MMRN1 (1)	acclimation
6	34.72	34.80	0.003960894	Krasnoyarsk	DCMS	1	MMRN1 (1)	acclimation
6	34.50	34.75	6.39E-06	Volgograd	DCMS	1	MMRN1 (1)	acclimation
6	33.28	34.60	0.001085822	GROUP2	hapFLK	1	CCSER1 (1)	feed intake
6	34.22	34.47	8.89E-05	Volgograd	DCMS	1	CCSER1 (1)	feed intake
6	33.33	34.26	0.000135783	All	hapFLK	1	CCSER1 (1)	feed intake
6	59.32	59.45	3.41E-09	Altai Mountain	DCMS	1	RBM47 (1)	growth
6	59.34	59.45	3.26E-06	Groznensk	DCMS	1	RBM47 (1)	growth
6	59.35	59.42	0.004758624	Kulundin	DCMS	1	RBM47 (1)	growth
6	59.36	59.41	0.001116515	Krasnoyarsk	DCMS	1	RBM47 (1)	growth
6	69.84	70.85	5.07E-08	All	hapFLK	4	KIT (1)	coat colour/domestication
6	69.66	70.59	0	GROUP1	hapFLK	3	PDGFRA (1) KIT (2)	acclimation, coat colour/domestication
6	115.08	115.56	0.003511895	GROUP2	hapFLK	5	ADD1 (1)	acclimation
7	20.98	21.22	2.53E-08	Karachaev	DCMS	11	ZFHX2 (1)	temperature sensation
7	20.96	21.11	8.00E-05	Buubei	DCMS	6	ZFHX2 (4)	temperature sensation
7	21.01	21.08	0.005941688	Lezgin	DCMS	1	ZFHX2 (1)	temperature sensation
7	21.00	21.04	5.71E-05	Krasnoyarsk	DCMS	1	ZFHX2 (1)	temperature sensation
7	21.01	21.04	0.000126518	Salsk	DCMS	1	ZFHX2 (1)	temperature sensation
7	21.01	21.04	0.006713729	Altai Mountain	DCMS	1	ZFHX2 (1)	temperature sensation
7	89.24	89.81	2.26E-05	GROUP1	hapFLK	4	TSHR (1)	acclimation
7	89.35	89.55	9.23E-08	Lezgin	DCMS	2	TSHR (2)	acclimation
7	89.39	89.48	0.004845986	Karakul	DCMS	1	TSHR (1)	acclimation
9	77.25	77.91	1.34E-07	Edillbai	DCMS	2	VPS13B (2)	milk
9	77.24	77.87	0.000648121	GROUP1	hapFLK	2	VPS13B (1)	milk
9	77.07	77.17	0.001546422	Karakul	DCMS	1	VPS13B (1)	milk
9	77.09	77.13	0.006246497	Salsk	DCMS	1	VPS13B (1)	milk
10	23.77	24.44	0.000617495	GROUP2	hapFLK	2	UFM1 (1)	Domestication
10	23.60	24.42	9.64E-05	All	hapFLK	3	UFM1 (1)	Domestication
10	23.90	24.31	0.007350694	GROUP1	hapFLK	1	UFM1 (1)	Domestication
10	25.16	30.13	0	All	hapFLK	18	RXFP2 (1) HSPH1 (4)	wool, horns, acclimation
10	28.03	30.10	0	GROUP2	hapFLK	11	RXFP2 (1) HSPH1 (4)	horns, acclimation
10	30.06	30.10	0.005654316	Baikal	DCMS	1	HSPH1 (1)	acclimation
10	28.16	30.00	0	GROUP1	hapFLK	10	RXFP2 (1)	horns
10	29.39	29.75	3.78E-12	Edillbai	DCMS	1	RXFP2 (1)	horns
10	29.36	29.64	2.56E-06	Russian Longhaired	DCMS	1	RXFP2 (1)	horns
10	29.32	29.63	1.23E-06	Romanov	DCMS	1	RXFP2 (1)	horns
10	29.47	29.63	1.14E-05	Karachaev	DCMS	1	RXFP2 (1)	horns
10	29.47	29.55	0.002108118	Groznensk	DCMS	1	RXFP2 (1)	horns
10	29.44	29.49	0.000484163	Altai Mountain	DCMS	1	RXFP2 (1)	horns
10	25.19	28.02	1.42E-07	GROUP2	hapFLK	7	NBEA (1)	wool
10	26.17	26.93	0.000427859	GROUP1	hapFLK	1	NBEA (1)	wool
10	26.55	26.66	0.001229851	Kulundin	DCMS	1	NBEA (1)	wool
10	26.30	26.56	0.000110212	Edillbai	DCMS	1	NBEA (1)	wool
10	26.33	26.41	0.000152487	Groznensk	DCMS	1	NBEA (1)	wool
10	26.15	26.17	0.001280272	Kulundin	DCMS	1	NBEA (1)	wool
10	26.15	26.17	0.009743166	Baikal	DCMS	1	NBEA (1)	wool
10	35.95	36.35	0.000164969	Tuva	DCMS	8	IFT88 (6)	reproduction
10	36.05	36.25	3.80E-05	Edillbai	DCMS	2	IFT88 (2)	reproduction
10	35.99	36.12	0.003724073	Romanov	DCMS	3	IFT88 (1)	reproduction
10	53.47	53.76	0.005873975	GROUP1	hapFLK	1	EDNRB (1)	acclimation
11	36.89	37.00	0.00235957	Volgograd	DCMS	1	B4GALNT2 (1)	reproduction
11	36.92	36.95	0.00700516	Groznensk	DCMS	1	B4GALNT2 (1)	reproduction
11	36.92	36.95	0.002079112	Karachaev	DCMS	1	B4GALNT2 (1)	reproduction
11	36.89	36.94	0.008582697	Karakul	DCMS	1	B4GALNT2 (1)	reproduction
13	41.71	41.86	1.37E-05	Groznensk	DCMS	4	ACSS1 (1)	milk
13	41.70	41.84	5.56E-07	Volgograd	DCMS	4	ACSS1 (1)	milk
13	41.72	41.83	7.68E-06	Altai Mountain	DCMS	3	ACSS1 (1)	milk
13	47.98	49.75	7.29E-11	All	hapFLK	3	PPP1CC (1) BMP2 (2)	body size, reproduction
13	48.34	49.40	1.74E-11	Karachaev	DCMS	3	PPP1CC (1) BMP2 (3)	body size, reproduction
13	48.43	49.30	9.62E-07	GROUP1	hapFLK	3	PPP1CC (1) BMP2 (3)	body size, reproduction
13	48.52	49.30	7.96E-09	Tuva	DCMS	2	PPP1CC (1)	reproduction
13	48.83	49.17	8.51E-11	Kulundin	DCMS	1	PPP1CC (1)	reproduction
13	48.80	49.15	3.36E-08	Salsk	DCMS	1	PPP1CC (1)	reproduction
13	48.84	49.15	5.59E-05	Volgograd	DCMS	1	PPP1CC (1)	reproduction
13	48.83	49.12	4.19E-05	Russian Longhaired	DCMS	1	PPP1CC (1)	reproduction
13	48.83	49.08	0.003588777	Groznensk	DCMS	1	PPP1CC (1)	reproduction
13	48.29	48.66	0.005674193	GROUP2	hapFLK	1	BMP2 (1)	body size
13	63.47	63.91	3.91E-06	Romanov	DCMS	10	ACSS2 (1)	milk
14	7.30	7.48	0.000183645	Karachaev	DCMS	2	GAN (1)	temperature sensation
14	13.59	15.49	0	GROUP1	hapFLK	30	MC1R (1)	coat colour/acclimation
14	13.75	14.80	0	All	hapFLK	22	MC1R (2)	coat colour/acclimation
14	14.15	14.43	2.15E-06	Karachaev	DCMS	8	MC1R (3)	coat colour/acclimation
14	14.14	14.27	1.18E-05	Karakul	DCMS	6	MC1R (3)	coat colour/acclimation
14	14.20	14.26	0.003770506	Lezgin	DCMS	5	MC1R (3)	coat colour/acclimation
14	14.14	14.25	8.10E-05	Tuva	DCMS	5	MC1R (4)	coat colour/acclimation
14	14.20	14.23	0.002109007	Baikal	DCMS	3	MC1R (2)	coat colour/acclimation
14	14.21	14.23	0.005865778	Altai Mountain	DCMS	2	MC1R (1)	coat colour/acclimation
14	54.32	54.41	7.27E-07	Volgograd	DCMS	3	MAMSTR (1)	lipid metabolosm
14	54.33	54.40	2.20E-05	Karachaev	DCMS	1	MAMSTR (1)	lipid metabolosm
14	54.34	54.40	5.36E-05	Baikal	DCMS	2	MAMSTR (1)	lipid metabolosm
14	54.31	54.39	0.000163454	Salsk	DCMS	4	MAMSTR (4)	lipid metabolosm
14	54.35	54.39	0.003248022	Groznensk	DCMS	1	MAMSTR (4)	lipid metabolosm
14	54.31	54.39	0.001065341	Kulundin	DCMS	4	MAMSTR (4)	lipid metabolosm
14	54.34	54.39	0.00166944	Altai Mountain	DCMS	1	MAMSTR (1)	lipid metabolosm
14	54.36	54.39	0.001659425	Romanov	DCMS	1	MAMSTR (1)	lipid metabolosm
15	17.37	17.43	0.001101253	Groznensk	DCMS	1	ATM (1)	milk
15	17.37	17.42	0.003777078	Baikal	DCMS	1	ATM (1)	milk
15	17.37	17.41	0.002123085	Salsk	DCMS	1	ATM (1)	milk
18	65.86	66.20	0.000807705	Tuva	DCMS	3	TECPR2 (1)	temperature sensation
18	65.89	66.04	0.003657536	Lezgin	DCMS	2	TECPR2 (2)	temperature sensation
19	0.85	0.89	0.006060865	Altai Mountain	DCMS	1	EGFR (1)	acclimation
19	6.84	6.88	0.002683568	Edillbai	DCMS	1	CMTM6 (1)	reproduction
19	6.83	6.85	0.006206755	Karakul	DCMS	1	CMTM6 (1)	reproduction
19	31.51	31.87	1.06E-06	GROUP1	hapFLK	1	MITF (1)	coat colour/domestication
19	31.51	31.86	0.000136197	All	hapFLK	1	MITF (1)	coat colour/domestication
19	31.60	31.65	0.002412391	Karakul	DCMS	1	MITF (1)	coat colour/domestication
20	34.19	34.23	0.003574509	Volgograd	DCMS	1	PRL (1)	acclimation
22	41.25	41.33	0.003671941	Buubei	DCMS	1	HTRA1 (1)	reproduction
22	41.23	41.31	0.00040073	Karakul	DCMS	1	HTRA1 (1)	reproduction
22	41.25	41.31	0.006635799	Tuva	DCMS	1	HTRA1 (1)	reproduction
23	25.42	26.46	0	GROUP1	hapFLK	12	DSG@ (1–4), DSC@ (7–9)	wool
23	26.20	26.36	1.05E-05	Russian Longhaired	DCMS	2	DSC@ (1–2)	wool
23	25.90	26.34	5.97E-05	All	hapFLK	5	DSG@ (1–2), DCG@ (3–5)	wool
23	25.90	26.33	8.02E-10	Altai Mountain	DCMS	6	DSG@ (1–3,5), DSC@ (4,6)	wool
23	25.98	26.11	0.002876356	Russian Longhaired	DCMS	3	DSG@ (−3)	wool
23	43.81	44.51	9.44E-06	Salsk	DCMS	3	MC5R (1) MC2R (3)	acclimation
23	43.75	44.06	0.000654721	Baikal	DCMS	3	MC2R (1) MC5R (2)	acclimation
25	18.69	18.88	0.001140569	GROUP1	hapFLK	2	EGR2 (1)	temperature sensation

We also found several genes that were previously proposed to be related to thermal adaptations [14]. Among them was the *EGFR*, a membrane receptor for epidermal growth factors and a top DCMS candidate in a 33 Kbp region on OAR19 reported for the Altai Mountain sheep breed from Siberia. According to Wollenberg Valero and co-workers (2014) *EGFR* could represent a functional hub for the relay of thermal signalling [14]. Another gene involved in adaptation to hot/cold environment was the heatshock protein H1 (*HSPH1*) [14], the top-ranked gene found in a DCMS-reported region on OAR10 for the Baikal sheep. Several genes linked to climate adaptation through their interaction with the *POMC* (pro-opiomelanocorin receptor), shown to be involved in energy homeostasis, melanocyte stimulation and immune response [14], were top-ranked in selected regions of Russian sheep breeds. Among them are melanocortin receptors: *MC1R*, *MC2R,* and *MC5R.* The *MC1R* was the top gene in a hapFLK reported region on OAR14 for GROUP1 and in a DCMS reported region for Altai Mountain sheep. The *MC2R* was the top gene for a DCMS-reported region on OAR23 found in Baikal breed. The *MC5R* was the top gene in region found by the DCMS method in the Salsk sheep. The melanocortin receptors are involved in the movement and positioning of melanocytes, suggesting their possible contribution to adaptation to different light regimes [14], however these genes could also be involved in formation of coat colours, suggesting their contribution to economically important traits [32]. Another gene, functionally linked to *POMC* [14] was the *MMRN1* with SNPs associated with winter duration in 54 human populations [33]. It was the top ranked gene in a hapFLK reported region on OAR6 for the all-breed analysis, and in the regions found by the DCMS method for Volgograd and Krasnoyarsk sheep.

We further found genes previously identified in a genome-wide scan for climate-mediated selective pressures in sheep [18] in the DCMS or hapFLK-reported regions. These included the *NMUR1*, *EDNRB*, and *PRL* all being the top genes in the DCMS/hapFLK reported intervals for the Romanov, GROUP1, and Volgograd breeds, respectively. The hapFLK method reported another region under putative selection for GROUP1 with *PDGFRA* found near the most significant SNP. Under cold stress, proliferation of endothelial cells and interstitial cells expressing *PDGFRA* increases 3-4 fold in brown adipose tissue; it is a key gene involved in cold-induced adipogenesis in mice [34]. Also, the adipocyte determination and differentiation-dependent factor 1 (*ADD1*) known to be involved in cold adaptation through brown adipocytes [35] was top-ranked for a region on OAR6 reported in GROUP2 by hapFLK. Consistent with the expected role of brown adipose tissue in adaptation to cold climate sheep breeds from Siberia: Kulundin, Altai Mountain, and Baikal all had a strong signature of selection (q-value ranges from 10^− 5^ to10^− 7^; DCMS) near the *ADAMTS5*, shown to be involved in the adiposity and metabolic health. Enhanced thermogenesis through the browning of white adipose tissue was reported in *ADAMTS5* knock out mice upon cold exposure [36, 37]. In accordance with the previous findings in Chinese native sheep [11] and indigenous Sunite sheep [38] we detected a signature of selection by DCMS near *TSHR* in Karakul and Lezgin breeds. *TSHR* was previously associated with metabolic regulation and photoperiod control or reproduction in vertebrates [11].

### Morphological traits and adaptations

Of the 3191 genomic intervals (3069 from DCMS and 122 from hapFLK analyses) under putative selection, 50.6% overlapped with regions previously predicted to have been under selection in sheep in different studies [1, 7, 11, 39] (see Additional file 2). Among these previously detected regions, strong signals of differentiation were obtained in the regions containing well known candidate genes related to morphology, adaptation, and domestication (e.g., *KITLG, KIT, MITF,* and *MC1R*), wool quality and quantity (e.g., DSG@, DSC@, and KRT@) growth and feed intake (*HOXA@, HOXC@, LCORL, NCAPG, LAP3,* and *CCSER1*), reproduction (*CMTM6, HTRA1, GNAQ, UBQLN1,* and *IFT88*), and milk traits (*ABCG2, SPP1, ACSS1,* and *ACSS2;* Fig. 2, Table 2).

#### Fleece related traits

Quantity and quality of wool is one of the most economically important traits in sheep. Consistent with this, the hapFLK analysis shows a strong signature of selection (q-value < 10^− 5^) in the group of all Russian breeds on OAR23 in the region containing a cluster of seven desmosomal genes (*DSG@* and *DSC@*). This selection signal becomes much stronger for GROUP1 (q-value < 10^− 23^), but the region is narrower and contains only the *DSG@* genes. DSMC splits this interval in two regions for the Russian Longhaired breed, suggesting separate selection sweeps near the DSG and DSC gene clusters. The Altai Mountain breed, however, exhibits the wider selection signal covering both clusters. The *DSG4* (*desmoglein 4*) is a known candidate for wool length and crimp in sheep [40], likely to be related to white and black coat colour in goats [41] and is a known cause of the recessive hairless phenotype in rats [42]. The *DSG1* and *DSG3* are associated with hair growth and follicle structure and the *DSC2* with woolly/straight hair phenotype in sheep [43, 44]. The DSMC method reports another strong selection signal (q-value < 10^− 7^) near the keratin gene cluster (KTR@) on OAR3 for the fine fleeced breeds: Baikal, Kulundin, Salsk, Krasnoyarsk, Groznensk, and Volgograd. For five out of the six breeds the selection intervals are relatively wide (> 100 Kbp) and contain multiple keratin genes while the Volgograd breed contains a narrower interval (~ 50 Kbp) with the *KRT72* gene only. Multiple members of the keratin cluster were shown to be related to fleece development. *KRT5* is related to fleece development and function [45], while *KRT71* is associated with the curly hair phenotype in sheep [46].

#### Coat colour

We identified a large group of known candidate genes related to coat colour, a trait of economic importance, also related to domestication and historical breed formation [47]. As expected, strong signatures of selection were found in the regions containing the genes *KIT* on OAR6, and *KITLG* on OAR3. The *KIT proto-oncogene receptor tyrosine kinase* (*KIT*) is associated with coat colour in various sheep breeds [5] and other species [48]. In our study, hapFLK identified the chromosomal interval containing this gene to be under strong selection in the set of all Russian breeds and GROUP1. *KITLG* is associated with pigmentation in sheep [49], roan coat phenotype in goats [50], and cattle [51]. The DCMS method reported this gene to be the top-ranked candidate in the selected regions of the Buubei and roan Edilbai breeds. Another gene found in a region of putative positive selection, the *melanocortin 1 receptor* (*MC1R*)*,* was top-ranked in the hapFLK-reported region in GROUP1 and in the DCMS interval identified in the Altai Mountain breed. *MC1R* has a pleiotropic effect, known, among other traits, to influence coat colour in sheep [52] and cattle [53]. The *microphthalmia transcription factor* (*MITF*) is a regulator of melanocyte development, and is associated with coat colour in mouse [54], dog [55], and other species. A strong signature of selection (q-value < 10^− 6^) was reported by hapFLK for GROUP1 in a ~ 350 Kbp interval on OAR19 containing *MITF,* and by DCMS for the Karakul breed. We identified an additional 20 genes in selected regions of Russian sheep breeds, reported by either hapFLK or DCMS (or both) methods that could be related to coat colour, fleece and other phenotypes in Russian sheep breeds (see Additional file 3).

#### Milk and lactation-related traits

The hapFLK method reported a large, ~ 5.7 Mbp region on OAR6 (34.63–40.33 Mbp) containing multiple candidate genes associated with milk production, growth, and feed efficiency (*MEPE, IBSP, SPP1, PKD2, ABCG2, LAP3, NCAPG, LCORL, FAM13A, FAM184B, DCAF16, HERC6,* and *SNCA*) for the GROUP2 set of breeds*.* A part of this region (37.25–37.45 Mbp) was also reported for GROUP1, including candidate genes *NCAPG, DCAF16, FAM184B.* A strong positive selection around the *ABCG2, SPP1, LAP3, NCAPG, LCORL*, *PKD2, IBSP,* and *MEPE* genes has been reported in domestic sheep including most European [1] and Chinese indigenous breeds [39]. A major milk-related trait gene, the *ATP-binding cassette, sub-family G (white), member 2* (*ABCG2* [56]) was found in intervals reported by DCMS for multiple Russian breeds but was not top-ranked in any of them. On the other hand, the region containing three genes *ABCG2*, *SPP1,* and *PKD2* was under selection in the Baikal sheep with *SPP1* (*osteopontin*) reported as the top-ranked gene by DCMS. *SPP1* is associated with milk protein percentage, milk yield, milk protein yield, and lactation regulation in dairy cattle [57]. Signatures of selection near the *SNCA*, potentially related to milk protein and fat traits in dairy cattle [58] were reported by DSMC for the Kulundin, Volgograd, Krasnoyarsk, and Baikal breeds. Two family members, the *ACSS1* and *ACSS2* previously associated with the mammary gland function and milk fatty acid composition in sheep [59, 60], cattle [61], and yaks [62] were the top-ranked genes in positively selected regions reported by DCMS. The *ACSS1* gene was reported for the Volgograd, Groznensk, and Altai Mountain, and *ACSS2* for the Romanov breeds. Another gene related to the mammary gland function was the *ATM* on OAR15, found in a DCMS-reported region for the Salsk, Baikal, and Groznensk breeds. The *ATM* contributes to mammary gland homeostasis and its knockout leads to a progressive lactation defect in mice [63]. In addition, we found *VPS13B,* a known candidate gene for milk-related traits in buffalo [64], in the interval reported by hapFLK for GROUP1. The DCMS analysis further reported this region to be under putative selection in the Karakul, Salsk, and Edilbai sheep.

#### Growth and feed intake

Two genomic regions containing clusters of HOX genes were reported by hapFLK for GROUP1. The first cluster contained the HOXC@ on OAR3 while the second, HOXA@ on OAR4. These regions were further confirmed to be under putative positive selection by the DCMS method. The region containing the HOXC@ could be under selection in the Lezgin, Edilbai, and Karakul breeds while the HOXA@-containing region, in the Karachaev, Kulundin, Russian Longhaired, Buubei, and Karakul breeds. The HOX genes are involved in regulation of limb development in mammals [65, 66], with the HOXA@ also being associated with body composition and structure in pigs [67] and HOXC@ being associated with tail fat deposition in sheep [68].

The region containing *LAP3*, *NCAPG, LCORL,* and *HERC6* genes on OAR6 is known to be related to growth traits, carcass composition, body size, weight and height in sheep [1], horses [69], and cattle [70, 71]. The results obtained using hapFLK and DCMS showed varying patterns of selective sweeps near these genes in Russian sheep breeds. Thus, according to hapFLK, *LCORL* was top-ranked for the all-breed analysis. This was further supported by DCMS for the Lezgin, Buubei, and Edilbai breeds while the *NCAPG* was top-ranked by hapFLK for the GROUP1 only. The *LAP3* from the same area was top-ranked for the Baikal and Altai Mountain breeds and the *HERC6* for Volgograd, Karakul, and Altai Mountain implying multiple signatures of selection in this region. The hapFLK approach reveals a ~ 1 Mbp interval containing only one gene, the *CCSER1,* on OAR6 in the all-breed set as well as in GROUP2, while DCMS further confirms this signature of selection in Volgograd, Krasnoyarsk, and Baikal breeds. Variants in the *CCSER1* are associated with the feed efficiency in beef cattle [72].

Furthermore, the DSMC method reported signatures of selection in regions near the functional gene candidates *RBM47*, *KCNE2*, *CPED1*, and *MAMSTR* in multiple sheep breeds. These candidate genes are responsible for processes related to growth (*RBM47* [73]), thyroid hormone biosynthesis (*KCNE2* [74]), development of bone mineral density (*CPED1* [75])*,* and lipid and glucose metabolism (*MAMSTR* [76])*.*

#### Reproduction

Both hapFLK and DCMS reported putative positive selection in multiple breeds for genomic regions containing genes with known effects on reproduction in mammals, including fertility: *DNAJB14* [77], gonad development and sperm maturation: *GNAQ* [78], spermatogenesis: *UBQLN1* [79], *IFT88* [80], and *PPP1CC* [81]. The DCMS method detected selection sweeps near *CMTM6* in Karakul and Edilbai sheep and near *HTRA1* in Karakul, Tuva, and Buubei sheep. These genes are associated with off-season reproduction traits, such as year-around oestrous behavior in sheep (*HTRA1* [82] and the evolution of sperm and the circadian rhythm systems in mammals (*CMTM6* [83]). The DCMS method further reported narrow selection sweeps for multiple Russian sheep breeds in the areas of *B4GALNT2* which was proposed as a strong candidate gene for ewe fertility [84].

## Discussion

Here we present the first comprehensive study of signatures of selection in the genomes of 15 native sheep breeds from the Russian Federation, for most of which we recently revealed the phylogenetic and population history in the context of related breeds from other countries [20]. In contrast to our phylogenetic study, based on moderate-density SNP array genotypes, genotyping for the present study was performed on a high-density array required to reveal the majority of selective sweeps. The analysis of the data was performed using complimentary approaches: the hapFLK and DCMS, which allowed us to detect signatures of selection that are putatively related to adaptation of breeds to local environments and human needs.

More than 50% of the putatively selected regions detected in the present study overlap with previously reported signatures of selection in various sheep breeds [1, 7, 11, 39], suggesting that our approach is robust enough to detect the expected signals of selection and that our dataset is different enough from the previously published ones to reveal new strong selective sweeps. The overlap observed between the hapFLK and the DCMS results point to putatively selected regions that are detectable using different models. The overlapping regions mainly contained known targets of selection in sheep. However, in general, hapFLK detects fewer but longer selected regions, while DSMC identifies a larger number of shorter intervals. This indicates that hapFLK could be more efficient in detecting regions with long haplotypes under selection, while DCMS can further dissect these intervals and point to specific genes under putative selection in individual breeds. DCMS is also capable of detecting shorter intervals, not found by hapFLK. This is confirmed by the fact that the region on OAR6, containing multiple genes related to milk production and growth traits was reported as a single interval by hapFLK but DCMS reported different regions within this interval containing different genes to be under selection in individual breeds. We detected the strongest signatures of selection shared between two groups of Russian sheep breeds in the regions of genes related to brain development, growth, milk production, and horned/polled phenotypes. These groups of genes are likely to be related to the process of domestication and historical breed formation.

In contrast to our recent study on the signatures of selection in Russian native cattle breeds [85] and high-density analysis of popular commercial breeds from Zhao and co-workers (2015) [86] we did not observe breeds where major milk-production related genes (e.g., *DGAT1* and *ABCG2*) would be reported as top ranked. This could be related to the fact that the sheep breeds used for milk production in the Russian Federation (e.g., Lezgin and Karachaev) cannot be considered as strictly dairy breeds. They are also breed for wool and meat, and these traits are often more important.

Consistent with this and our previous publication [20], the PCA and ADMIXTURE analysis separated the Russian sheep breeds into two major clusters which follow wool quality type [20]. The DCMS method further suggested that the cluster of KRT genes on OAR3 is under positive selection in fine wool breeds, confirming that the keratin genes could be related to the quality of wool, as it has been shown in other studies [46]. Interestingly, the region containing a cluster of desmosomal genes was under strong selection in coarse wool breeds (GROUP1). The DCMS approach, however, points to two breeds from GROUP2, which also had this region under putative selection. GROUP2 includes breeds with the fine and semi-fine wool. According to DCMS the desmosomal genes were under selection only in the semi-fine Russian Longhaired and Altai Mountain breeds. Combined with the hapFLK results, this may indicate that selection in desmosomal genes could be related to ‘coarseness’ of the sheep wool but this hypothesis needs further verification using sequenced genomes from breeds of all the three types.

Unlike the Yakut cattle [87], domestic sheep cannot be normally found above the Polar Circle in Russia. However, the harsh environments of Siberia and other parts of the Russian Federation still impose significant selective pressure on local sheep breeds. Consistent with this, multiple genes related to acclimatization were found in putatively selected regions in Russian sheep breeds. In another study [85] we found one of the strongest signals of selection in the Yakut cattle, adapted to survive at − 50 °C, in the area of the gene *RETREG1,* one of the key genes involved in the hereditary sensory and autonomic neuropathy, type II in humans [27]. This disorder in humans is accompanied by an inability to feel pain and low temperatures. In the present study, we identified multiple genes with known key contributions to human hereditary sensory and autonomic neuropathies (types I-IV) in selected regions of individual or multiple sheep breeds. It is tempting to hypothesise that changes within these genes could be related to adaptation to the harsh environments of the Russian Federation. For the breeds from Siberia, changes in these genes could be beneficial for surviving cold winters, but in other breeds they could be advantageous for survival in cold mountain climates. However, a definite answer to this question could only be obtained after the actual sequences of these genes are obtained for breeds dwelling in different environments and the presence and frequencies of missense or regulatory variants are compared.

Similar to Russian native cattle breeds, sheep breeds express signatures of selection in regions of genes related to brown adipogenesis. Brown adipose tissue is an important organ involved in in non-shivering thermogenesis indicating that these genes could be related to adaptation to cold climates. However, the adipose tissue contributes to phenotypic differences between breeds (fat- and thin-tailed sheep) and to meat quality, which is an economically important trait. This suggests that more studies need to be done to distinguish potential adaptive effects of the genes involved into adipogenesis, from their contribution to economically important traits in sheep and cattle.

## Conclusions

In conclusion, we identified signatures of selection in Russian local sheep breeds of European and Asian origin. These signatures point to known regions related to economically important traits, domestication and breed formation as well as to intervals of the sheep genome that could contribute to adaptation of breeds to their corresponding local environments. Our results indicate that a detailed study(ies) of Russian local breeds involving whole-genome sequencing should focus on identifying causative genetic variants or haplotypes. These polymorphisms should be in turn the focus of future efforts on the improvement of local breeds, or for selecting multinational commercial breeds which would be better suited for the environments of the Russian Federation and Northern Eurasia.

## Methods

### Sample collection

Tissue samples for the Lezgin, Karachaev, Karakul, Edilbai, Romanov, Russian Longhaired, Groznensk, Salsk, and Volgograd breeds and blood samples for the Buubei, Tuva, Altai Mountain, Krasnoyarsk, Baikal, and Kulundin breeds were collected from farms and breeding centres across Russia. All samples were collected by trained personnel following strict veterinary regulations. We studied the pedigrees of animals to avoid sampling of close relatives (siblings, parents, and offspring). Tissues and blood were stored at -80 °C until use.

### Genotyping of Russian sheep breeds

DNA from tissue samples of nine sheep breeds was extracted using Nexttec columns (Nexttec Biotechnology GmbH, Germany) following the manufacturer’s instructions. DNA from blood samples of six additional sheep breeds was extracted using cell lysation followed by phenol-chloroform extraction [88]. DNA samples of all breeds were genotyped using the Ovine Infinium® HD SNP BeadChip (600 K SNPs) to produce dense genome coverage. Genotypes were called using GenomeStudio 2 software (Illumina, San Diego, USA) and samples with overall calling rate < 95% were removed from further analyses. The produced files with genotypes (.ped) and chromosomal positions (.map) were processed using PLINK v.1.90 whole genome analysis toolkit [89].

### Analysis of groups of populations

Divergence between populations can influence methods which assume a certain population structure and low level of genetic differentiation [7]. To reduce the effect of population structure on the hapFLK analysis, we performed the Principal Component Analysis (PCA [90]) and ADMIXTURE genetic clustering on all the studied breeds. Prior to the analyses, to reduce effects of linkage-disequilibrium between loci we pruned the dataset using the PLINK function --indep-pairwise 50 10 0.1 using only autosomal genotypes resulting in 95,809 variants for 312 animals. Then we ran ADMIXTURE for K = 1–20 and calculated cross-validation error for each run (*-cv*). The results of the ADMIXTURE analysis were visualized with PONG software [91] the results of PCA analysis were plotted within the *R* environment.

### Identification of signatures of selection with hapFLK statistics

We performed a genome scan for selective sweeps within each group of breeds and for all breeds simultaneously to infer regions which were under selection during the group/breed formation and in the ancestral population of all studied breeds using a haplotype-based statistics hapFLK [92]. Due to the hapFLK model assuming that selection acts on shared ancestral SNP allele frequencies, we excluded rare SNPs with low minor allele frequencies (MAFs) from each of the breed groups (MAF < 0.05). We also excluded poorly genotyped individuals (< 95% of SNPs with genotypes), loci genotyped in < 99% of samples, SNPs without chromosomal assignments, and SNPs on sex chromosomes in PLINK, using the commands: --maf 0.05, --mind 0.05, --geno 0.01, and --chr 1–26 prior to performing the genome selection scans. This resulted in 506,343 autosomal SNPs for the all-breed analysis, 492,607 and 499,219 for GROUP1 and GROUP2, respectively.

The hapFLK method takes the haplotype structure of the population into account. What was important for our dataset is that this method can account for population bottlenecks and migration. Reynolds distances and a kinship matrix were calculated by the hapFLK program v.1.4 [92]. For the hapFLK analysis, the number of haplotype clusters for each breed group were estimated with fastPhase [93] and were set as *-K* 40, 25, 35 for the all-breed set, GROUP1, and GROUP2, respectively. The expected maximum number of iterations was set to 30 for three groups. We applied midpoint rooting to all sets of breeds.

#### *P*-value calculation

For hapFLK, the calculation of raw *p*-values was performed assuming that the selected regions represent only a small fraction of the genome [7]. The genome-wide distribution of hapFLK statistics could be modelled relatively well with a normal distribution except for a small fraction of outliers from potentially selected regions [7]. Robust estimations of the mean and variance of the hapFLK statistic were obtained using the *R* MASS package *rlm* function to eliminate influence of outlying regions following Biotard and co-workers (2016) [94]. This has been done for each group (all breeds, GROUP1, and GROUP2). The hapFLK values were Z-transformed using these parameter estimates, and *p*-values were calculated from the normal distribution in *R*. The *R qvalue* package was used to correct *p*-values for multiple testing [95].

### Composite measure of selection (DCMS statistics)

Recent studies demonstrated the high efficiency of composite measures of selection over the single-statistic tests or their simple meta-analysis [96, 97]. Composite measures of selection such as de-correlated composite of multiple signals DCMS [96] allow more pricisely locate the selection signal and filter out spurious results specific for some methods. For this study we combined five well-established genome-wide statististics into a single DCMS framework [96]. The DCMS works by combining *p*-values from different statistics at each locus and correcting for the overall correlation between the statistics based on the covariance matrix. We aggregated the following statistics in the present work: haplotype homozygosity (H1 [98]), modified haplotype homozygosity statistics (H12 [98], fixation index (*F*_*ST*_ [99], Tajima’s D index [100] and nucleotide diversity (Pi [101]).

#### Haplotype-based statistics

Autosomal genotypes were phased using SHAPEIT2 software [102] with 400 conditioning states (-states 400) and effective population size parameter equal to 3000 (-effective-size 3000) as a safe estimate of genetic variation within our diverse dataset. The recombination rate along the chromosomes was corrected with a high-resolution ovine genetic map [103].

To calculate the haplotype-based H1 and H12 statistics the phased VCF file was converted to the format required by the H12_H2H1.py script (https://github.com/ngarud/SelectionHapStats) from Garud and co-workers (2015) [98]. The statistics were calculated for each autosome of each breed using overlapping windows of 25 single nucleotide variants (SNVs, -w 25) with the step size equal to 1 (-j 1) and allowing zero false-positive SNVs per window (-d 0).

#### Tajima’s D statistics

To calculate Tajima’s D statistics, we first formed chromosome intervals based on the output of the H1 statistics and then passed them to the *bcftools* (*view*) software [104] along with the breed-specific gzipped VCF file, before being piped to the vcftools -TajimaD function. The work was performed in a parallel mode with assistance of GNU PARALLEL [105] to reduce calculation time.

#### Fixation index (F_ST_)

To quantify the population differentiation for every SNV we calculated *F*_*ST*_ index for each breed against the combined pooled sample of all other breeds using the plink --fst function. Negative *F*_*ST*_ values were converted into zeros and the statistic was smoothed for each chromosome using R *runmed* function in windows of 31 SNPs (k = 31, endrule = “constant”) to reduce noise.

#### Nucleotide diversity (pi)

Nucleotide diversity was calculated using the vcftools -site-pi option for each position and breed separately. To reduce the overall noise the statistic was smoothed with the *R runmed* function with a window size of 31 SNPs (k = 31, endrule = “constant”).

#### De-correlated composite of multiple signals (DCMS)

At the first step, we combined all the calculated statistics (H12, H1, Pi, Tajima’s D, *F*_*ST*_) into a single spreadsheet based on the SNV name. We then calculated genome-wide rank-based *p*-values for each statistic (stat_to_pvalue MINOTAUR function) using one-tailed tests (Pi and Tajima D – left-tailed; H1, H12, and *F*_*ST*_ – right-tailed) of the *R* MINOTAUR package [106]. To adjust for the correlation among the statistics we calculated the covariance matrix based on 300,000 randomly sampled SNPs using the *CovNAMcd* function with alpha = 0.75. The matrix was then used to calculate the DCMS statistic using *DCMS* function of the MINOTAUR package. We fitted the resulting DCMS statistics for each breed into a normal distribution using the robust fitting of the linear model method implemented in the *rlm R* function of the MASS package [94]. The fitted DCMS statistics were then converted into *p*-values using the *pnorm* function (lower.tail = FALSE, log.p = FALSE) and the *p*-values were finally converted to the corresponding q-values using the *qvalue R* function [95].

### Identification of chromosome intervals under selection and candidate genes

We downloaded the ovine gene annotations from the Biomart [107] which correspond to the Oar_v3.1 genome assembly [108]. Next, we considered chromosome intervals with SNPs with adjusted *p*-values < 0.01 to determine putative regions under selection. The boundaries of each interval were defined by the locations of the first flanking SNPs exhibiting adjusted *p*-values > 0.1. Within the selected intervals, genes were identified within 1σ value from the most significant SNP based on statistical value (DCMS or hapFLK) distribution similar to Fariello and co-workers [7]. This approach helps to balance the number of candidate genes reported between the “sharp” selection peaks, and intervals with many SNPs exhibiting similar statistics values where larger numbers of genes were reported. Finally, the genes were ranked based on their distance from the SNP with the highest statistics value in each region with larger ranks assigned to more distant genes.

## Additional files

Additional file 1: Overlapping regions under putative selection detected by both the DCMS and hapFLK approaches. (XLSX 106 kb)

Additional file 2: Putatively selected regions in genomes of 15 Russian sheep breeds as detected by the hapFLK and DCMS approaches. (XLSX 300 kb)

Additional file 3: Additional candidate genes top-ranked in selected regions of 15 Russian sheep breeds. (XLSX 11 kb)
